# Efficacy and safety of tacrolimus in the treatment of interstitial pneumonia with autoimmune features: a retrospective study

**DOI:** 10.3389/fphar.2025.1721639

**Published:** 2025-11-20

**Authors:** Yanzao Zhao, Xuemei Huang, Xinyue Cao, Jie Luo, Xi He, Qianhui Yang, Anji Xiong

**Affiliations:** 1 Department of Rheumatology and Immunology, The Affiliated Hospital of Southwest Medical University, Luzhou, Sichuan, China; 2 Department of Rheumatology and Immunology, The first people’s Hospital of Neijiang, Neijiang, Sichuan, China; 3 Department of Rheumatology and Immunology, Nanchong Central Hospital, The Affiliated Nanchong Central Hospital of North Sichuan Medical College, Nanchong Hospital of Beijing Anzhen Hospital Capital Medical University, Nanchong, Sichuan, China

**Keywords:** interstitial lung disease, autoimmune disease, tacrolimus, interstitial pneumonia with autoimmune features, treatment

## Abstract

**Objective:**

To evaluate the efficacy and safety of tacrolimus in interstitial pneumonia with autoimmune features (IPAF).

**Methods:**

48 IPAF patients receiving tacrolimus were retrospectively analyzed. Changes in high-resolution computed tomography (HRCT) scores, modified Medical Research Council (mMRC) dyspnea grade, inflammatory markers, and methylprednisolone dosage were assessed.

**Results:**

Tacrolimus treatment led to significant reductions in total HRCT score (P < 0.001), consolidation (P < 0.001), ground-glass opacity (GGO, P = 0.002), mMRC grade (P < 0.001), erythrocyte sedimentation rate (P < 0.001), C-reactive protein (P < 0.001), and methylprednisolone dosage (P < 0.001). Improvements in total HRCT score correlated with reductions in consolidation (ρ = 0.487, P < 0.001), GGO (ρ = 0.442, P = 0.002). Baseline consolidation score independently predicted imaging improvement (B = −0.249, p = 0.005). Patients with myositis-specific antibodies (MSAs, n = 28) showed broader improvement across all parameters than those with myositis-associated antibodies (MAAs, n = 15). Hypomagnesemia was the most common adverse event and was manageable.

**Conclusion:**

The results of this study demonstrate that tacrolimus significantly improves pulmonary inflammatory lesions, dyspnea symptoms, systemic inflammation levels, and facilitates steroid dose reduction in IPAF patients. Patients with baseline imaging features predominantly characterized by GGO and consolidation, or those positive for MSAs, may derive greater benefit from tacrolimus treatment. Further large-scale randomized controlled trials are warranted to validate its efficacy and safety.

## 1 Introduction

Interstitial lung disease (ILD) represents a heterogeneous group of diffuse parenchymal lung diseases characterized by high morbidity and mortality, featuring varying degrees of inflammation and/or fibrosis in the pulmonary interstitium, ultimately leading to parenchymal damage (Antoniou et al., 2014). ILD can be classified into ILD of known etiology, idiopathic interstitial pneumonia (IIP), granulomatous ILD, and unclassifiable ILD. Many patients with IIP exhibit clinical features suggestive of an autoimmune process (Bw et al., 2007; Fischer et al., 2010; Corte et al., 2012; Vij et al., 2011), but do not meet the diagnostic criteria for any known connective tissue disease (CTD). Therefore, in 2015, the European Respiratory Society/American Thoracic Society (ERS/ATS) proposed the term “interstitial pneumonia with autoimmune features” (IPAF) to describe these patients (Fischer et al., 2015). This terminology provides a unified platform for the management and observational study of such patients but does not offer specific treatment recommendations or clinical management guidelines.

IPAF shares characteristics between idiopathic pulmonary fibrosis (IPF) and CTD-ILD (Fernandes et al., 2019; Lee and Fischer, 2019; Oldham and Danoff, 2019). Clinically, CTD-ILD patients are primarily treated with a combination of immunosuppressants and corticosteroids (Kondoh et al., 2021); IPF patients benefit from antifibrotic therapy, while immunosuppressive therapy may increase disease risk (Travis et al., 2013). Currently, IPAF patients are mainly treated with immunosuppressive therapy and/or antifibrotic agents. The former includes glucocorticoids, mycophenolate mofetil (MMF), azathioprine, methotrexate, cyclophosphamide, tacrolimus, and rituximab; the latter includes pirfenidone and nintedanib (Bosch et al., 2022; D’Silva et al., 2021).

In the treatment of CTD-ILD, the combination of tacrolimus—a T-cell-specific inhibitor widely used in organ transplantation and various immune system diseases—and glucocorticoids has shown better prognosis compared to cyclosporine in treating dermatomyositis/polymyositis -ILD (Takada, Nagasaka, and Miyasaka, 2005), potentially improving short-term mortality with a manageable safety profile (Takada et al., 2020). It is also well-tolerated and effective in treating anti–aminoacyl–transfer RNA synthetase–associated ILD and idiopathic inflammatory myopathy (IIM) (Wilkes et al., 2005). In patients with acute exacerbation of IPF, tacrolimus combined with glucocorticoids effectively reduces mortality and leads to clinical improvement (Horita et al., 2011).

Despite these data, the efficacy of tacrolimus in IPAF remains undefined. We therefore performed a retrospective cohort study to evaluate the efficacy and safety of tacrolimus combined with glucocorticoids in the treatment of IPAF, primarily based on changes in high-resolution computed tomography (HRCT), thereby providing a reference for the clinical management of IPAF.

## 2 Materials and methods

### 2.1 Study design and study population

This was a retrospective observational study designed to evaluate the efficacy and safety of tacrolimus in patients with IPAF. We retrospectively screened the medical records of 271 patients with interstitial pneumonia who were hospitalized in the Department of Rheumatology and Immunology at Nanchong Hospital, Capital Medical University Affiliated Anzhen Hospital, between 2021 and 2025. Ultimately, 48 patients who met the diagnostic criteria for IPAF (Fischer et al., 2015) and had received tacrolimus treatment for at least 3 months were included. All enrolled patients were aged ≥18 years. For patients with a radiologic UIP pattern, the diagnosis of IPAF was established based on their concurrent fulfillment of serological and/or clinical domain criteria, thereby distinguishing them from IPF.

### 2.2 Data collection and variable definitions

The primary exposure of interest was tacrolimus. Exposure started and end dates were recorded using electronic health record prescription data. Additional clinical data extracted for analysis included age, sex, and smoking status. Clinical features encompassed inflammatory arthritis, Raynaud’s phenomenon, mechanic’s hands/finger ulcers, telangiectasia, finger edema, serositis, and extensor surface rash. Laboratory data collected consisted of complete blood count, complement levels, serum creatinine, inflammatory markers, ANA, anti-Jo1, anti-cyclic citrullinated peptide antibody, anti-dsDNA, anti-MDA5, anti-PM-Scl, rheumatoid factor, anti-ribonucleoprotein antibody, anti-Scl70, anti-Smith antibody, anti-SSA/SSB antibody, and other tRNA antibodies. Based on antibody profiles, patients were categorized into two groups: those with myositis-specific antibodies (MSAs) (including Jo-1, PL7, PL12, EJ, OJ, Mi-2, SRP, NXP2, TIF1γ, SAE, MDA-5, SC, JS, YRS, Zo, and HMGR) were assigned to the MSAs group, while those with myositis-associated antibodies (MAAs) (including SSA60, SSA52, RNP [U1, U2, and U3 subtypes], Ku, and Pm/Scl) were assigned to the MAAs group.

### 2.3 Tacrolimus treatment

The initial phase involved oral administration of tacrolimus at 1 mg once daily or 1 mg every 12 h, followed by close monitoring of tacrolimus trough blood concentrations. Given the variability in absorption rates and bioavailability among individuals, and considering that the optimal therapeutic trough concentration varies from patient to patient, dosages were adjusted based on individual patient conditions and disease progression. Dose escalation was discontinued once clinical improvement was observed. We aimed to maintain trough concentrations below 10 ng/mL. The actual median tacrolimus trough concentration in this study population was 5.10 (IQR: 2.88, 9.99) ng/mL. Tacrolimus trough levels were measured using a microparticle enzyme immunoassay. During tacrolimus treatment, Methylprednisolone (MPRED) dosages were adjusted by physicians according to the patient’s clinical status.

### 2.4 Treatment response assessment

The primary efficacy endpoint was the total score on high-resolution computed tomography (HRCT) of the chest. Secondary endpoints included the modified Medical Research Council (mMRC) dyspnea scale score, and inflammatory markers: C-reactive protein (CRP) and erythrocyte sedimentation rate (ESR). Efficacy was assessed based on changes between baseline and the last follow-up: baseline was defined as data collected within 3 months prior to initiating tacrolimus treatment, and the last follow-up was defined as the most recent complete follow-up data available during treatment.

#### 2.4.1 HRCT visual scoring method

As there is no unified HRCT scoring system for IPAF, and considering that IPAF and systemic sclerosis-associated interstitial lung disease (SSc-ILD) are both immune-mediated interstitial lung diseases with significant overlap in imaging, histopathology, and pulmonary function, (Fischer et al., 2008), this study adopted a modified Warrick-Lynch visual semi-quantitative scoring method for SSc-ILD as proposed by Goldin et al. (2008). Two senior radiologists, blinded to clinical information other than the IPAF diagnosis, independently interpreted the images and assigned scores. Scoring items included total disease extent, extent of fibrosis, extent of ground-glass opacity (GGO), extent of consolidation and extent of honeycomb cysts (HCs). If the difference between consecutive scores was ≥25% or the categorical score differed by more than one grade, a third radiologist was consulted to reach a consensus.

The scoring method was as follows: each lung was divided into three regions: upper (lung apex to aortic arch), middle (aortic arch to inferior pulmonary vein), and lower (inferior pulmonary vein to lung base). Each of the six regions was scored on a scale of 0–4 for the extent of lung abnormalities: 0, absent; 1, 1%–25%; 2, 26%–50%; 3, 51%–75%; and 4, 76%–100%. The following HRCT scores were recorded: GGO, fibrotic shadows, HCs, and consolidation. The total HRCT score was the sum of the scores for these four patterns. The definitions of these four patterns were based on the terminology from the Fleischner Society glossary of thoracic imaging terms (Bankier et al., 2024).

Chest imaging patterns were classified according to the international multidisciplinary consensus classification of idiopathic interstitial pneumonias proposed by the ERS/ATS (American Thoracic Society/European Respiratory Society International Multidisciplinary Consensus Classification of the Idiopathic Interstitial Pneumonias, 2002) into the following categories: usual interstitial pneumonia (UIP), nonspecific interstitial pneumonia (NSIP), organizing pneumonia (OP), NSIP with OP overlap (NSIP + OP), and lymphocytic interstitial pneumonitis (LIP).

#### 2.4.2 Modified Medical Research Council (mMRC)

The severity of dyspnea was assessed using the modified Medical Research Council (mMRC) dyspnea grade (Bestall et al., 1999). This scale grades breathlessness related to daily activities from 0 to 4, with higher grades indicating more severe dyspnea. Assessments were based on retrospective review of changes in condition documented in inpatient or outpatient medical records. The specific grades are: Grade 0: Breathless only with strenuous exercise. Grade 1: Short of breath when hurrying on level ground or walking up a slight hill. Grade 2: Walks slower than people of the same age on level ground because of breathlessness, or has to stop for breath when walking at own pace on level ground. Grade 3: Stops for breath after walking about 100 yards (approximately 91 m) or after a few minutes on level ground. Grade 4: Too breathless to leave the house or breathless when dressing or undressing.

### 2.5 Ethical statement

This study was approved by the Ethics Committee of Nanchong Hospital, Capital Medical University Affiliated Anzhen Hospital (Approval No.: 2025–150). The requirement for informed consent was waived. All patient data were anonymized during collection and handled in accordance with the ethical principles of the Declaration of Helsinki.

### 2.6 Statistical analysis

Statistical analyses were performed using SPSS version 27 and GraphPad Prism version 10. Categorical variables are presented as numbers (percentages) [n (%)], and comparisons between groups were performed using the chi-square test (or Fisher’s exact test when expected frequencies were <5). The normality of continuous variables was assessed using the Shapiro-Wilk test. Although a few indicators showed normal distribution for pre- and post-treatment measurements separately, their differences did not follow a normal distribution; the remaining indicators were non-normally distributed. Therefore, all continuous variables are expressed as median (interquartile range). Comparisons before and after treatment were analyzed using the Wilcoxon signed-rank test, with effect sizes (r values) reported. Box plots were generated to illustrate the distribution of each indicator before and after treatment. For comparisons between different groups (e.g., baseline characteristics or efficacy outcomes between groups), the Mann-Whitney U test was employed. Spearman’s rank correlation was used for correlation analysis, and correlation heatmaps were generated using GraphPad Prism 10. Multiple linear regression analysis was conducted to examine the influence of baseline indicators on treatment response. A P-value <0.05 was considered statistically significant.

## 3 Results

### 3.1 Baseline characteristics

A total of 48 patients meeting the diagnostic criteria for IPAF were included in this study. As summarized in Table 1, the baseline characteristics were as follows: the median age was 56 years (IQR: 50–59), with a predominance of female patients (34, 71%). Three patients had a history of smoking. Regarding the IPAF diagnostic domains, the most common clinical manifestation was inflammatory arthritis or morning stiffness (6, 12.5%), followed by Raynaud’s phenomenon (4, 8.3%). In the serological domain, anti-Ro (SS-A) antibody positivity was most frequently observed (26, 54.2%), followed by anti-tRNA synthetase antibodies (20, 41.7%) and ANA ≥1:320 (13, 27.1%). Morphologically, the predominant HRCT patterns were NSIP (23, 47.9%), NSIP with OP overlap (15, 31.3%), and UIP (9, 18.8%). Multi-compartment involvement was common (23, 47.9%), mainly presenting as unexplained pleural abnormalities and pericardial effusion (9, 18.8%). The most frequent combination of diagnostic domains was “serological + morphological” (38, 79.2%), followed by fulfillment of all three domains (10, 20.8%). None of the patients met the diagnostic criteria based solely on the “clinical + serological” combination.

**TABLE 1:** Baseline demographics and clinical data.

Baseline characteristic (n = 48)	Finding
Age, years, median (IQR)	54.90 ± 10.36
Male, n (%)	14 (29)
Female, n (%)	34 (71)
Smoking history	3/48
Frequency of positive clinical domain finding	
Distal digital fissuring (i.e.,“mechanic hands”)	0
Distal digital tip ulceration	0
Inflammatory arthritis or polyarticular morning joint stiffness >60 min	6/48
Palmar telangiectasia	0
Raynaud’s phenomenon	4/48
Unexplained digital oedema	1/48
Unexplained fixed rash on the digital extensor surfaces (Gottron’s sign)	1/48
Frequency of positive serologic domain finding	
ANA >1:320 titre, diffuse, speckled, homogeneous patterns ora.ANA nucleolar pattern [any titre] orb.ANA centromere pattern [any titre]	13/48
Rheumatoid factor >2x upper limit of normal	3/48
Anti-CCP	3/48
Anti-dsDNA	0
Anti-Ro (SS-A)	26/48
Anti-La(SS-B)	2/48
Anti-ribonucleoprotein (RNP)	1/48
Anti-SmithaAnti-topoisomerase (Scl-70)	2/48
Anti-tRNA synthetase (e.g., Jo-1, PL-7, PL-12; others are EJ, OJ, KS, Zo, tRS)	20/48
Anti-PM-Scl	3/48
Anti-MDA-5	1/48
Frequency of positive morphologic domain finding	
HRCT pattern	
NSIP	23/48
OP	1/48
NSIP with OP overlap	15/48
LIP	0
UIP	9/48
Multi-compartment involvement (in addition to interstitial pneumonia)	
Unexplained pleural effusion or thickening	23/48
Unexplained pericardial effusion	9/48
Unexplained intrinsic airways disease	3/48
Unexplained pulmonary vasculopathy	5/48
Diagnostic domains met	
Clinical and serological	0
Clinical and morphological	1/48
Serological and morphological	38/48
All three	10/48
Concomitant medications	
Pirfenidone	15/48
Nintedanib	0

Variables were presented as mean ± SD, or N (%). ANA, antinuclear antibody; CCP, cyclic citrullinated peptide; HRCT, high-resolution computed tomography; IPAF, interstitial pneumonia with autoimmune features; LIP, lymphocytic interstitial pneumonia; NSIP, nonspecific interstitial pneumonia; OP, organizing pneumonia; RF, rheumatoid factor.

### 3.2 Changes in outcome measures before and after treatment

**FIGURE 1:**
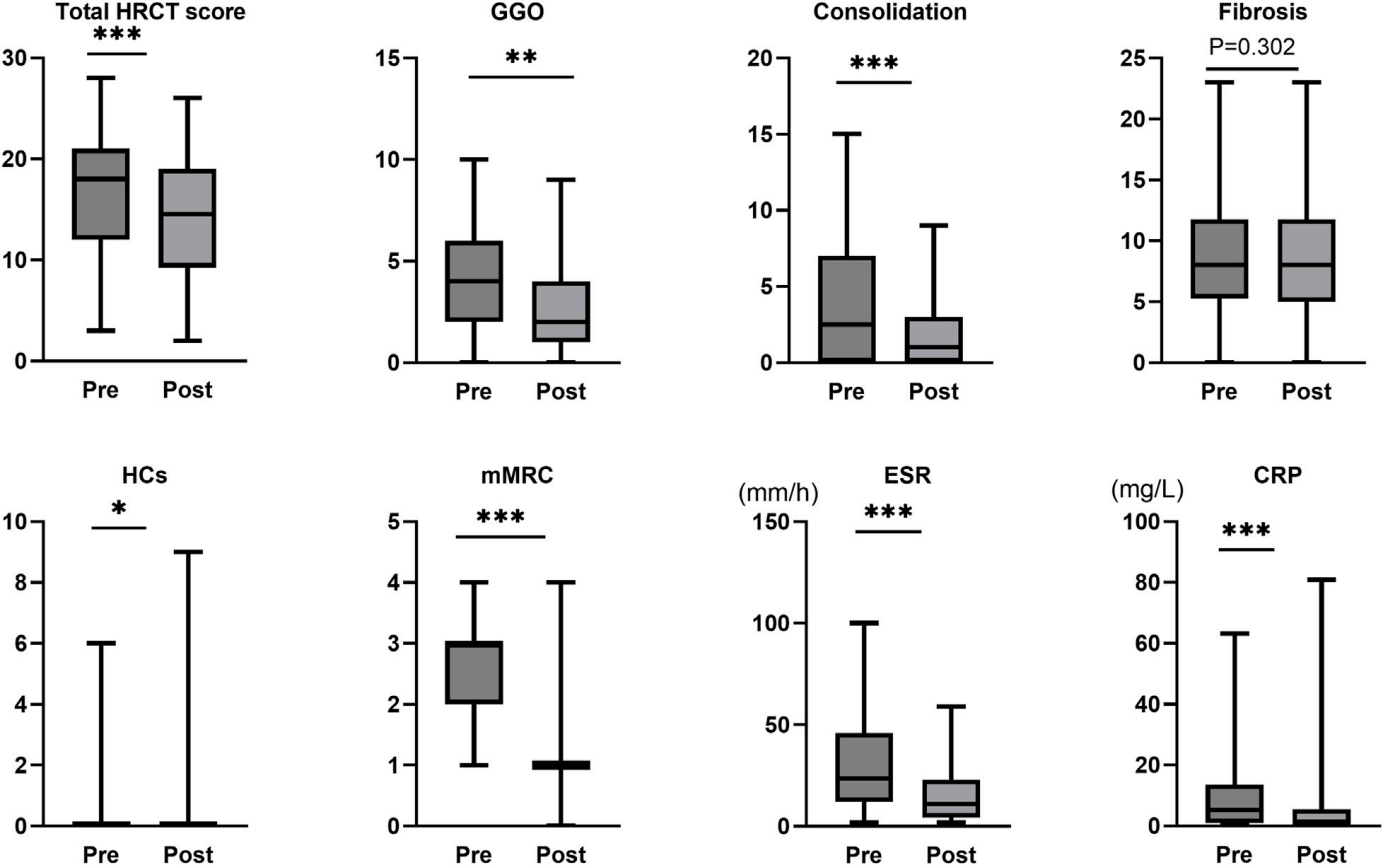
Assessment of treatment response to tacrolimus in IPAF. mMRC, modified Medical Research Council dyspnea scale; ESR, erythrocyte sedimentation rate; CRP, C-reactive protein; GGO, ground-glass opacity; HRCT, high-resolution computed tomography; Pre represents the status before tacrolimus treatment initiation, while Post represents the status after treatment. ***, P < 0.001; **, *P* < 0.01; *, *P* < 0.05.

Following tacrolimus treatment, significant improvements were observed in multiple key clinical and imaging parameters among IPAF patients compared to baseline (Wilcoxon signed-rank test). Radiologically, the total HRCT score of the lungs decreased significantly (median difference: −2, 95% Boot CI: −3 ∼ −1, P < 0.001), with the reduction primarily driven by the resolution of consolidation (median difference: −1, P < 0.001, r = 0.664) and ground-glass opacity (GGO) (median difference: −1, P = 0.002, r = 0.452). In contrast, the extent of pulmonary fibrosis showed no statistically significant change before and after treatment (median difference: 0, P = 0.302). Notably, although the median score for honeycombing remained 0 both pre- and post-treatment (median difference: 0), the change reached statistical significance (P = 0.026, r = 0.322). Individual patient analysis revealed that 7 out of 48 patients (14.6%) exhibited an increase in HCs extent, suggesting heterogeneity in treatment response. Regarding clinical symptoms and inflammatory markers, the dyspnea grade (mMRC) improved significantly (median difference: −1.5, P < 0.001, r = 0.768). Systemic inflammation markers, including ESR (median difference: −8, P < 0.001, r = 0.718) and CRP (median difference: −2.2, P < 0.001, r = 0.606), also demonstrated significant reductions. Additionally, the required dosage of MPRED was successfully tapered (median difference: −5, P < 0.001, r = 0.680) (Table 2; Figure 1).

**TABLE 2:** Comparison of clinical parameters in IPAF patients before and after tacrolimus therapy (n = 48).

Variable	Baseline median (IQR)	Post-treatment median (IQR)	Median change (95% Boot; CI)	p value
Total HRCT score	18 (12, 21)	14.5 (9.25, 19)	−2 (−3, −1)	<0.001
GGO	4 (2, 6)	2 (1, 4)	−1 (−1, 0)	0.002
Fibrosis	8 (5.25, 11.75)	8 (5, 11.75)	0 (−0.5, 0)	0.302
HCs	0 (0, 0)	0 (0, 0)	0 (0, 0)	0.026
Consolidation	2.5 (0, 7)	1 (0, 3)	−1 (−2, 0)	<0.001
mMRC	3 (2,3)	1 (1, 1)	−1.5 (−2, −1)	<0.001
ESR (mm/h)	23.5 (12, 46)	11 (4.25, 23)	−8 (−12, −6)	<0.001
CRP (mg/L)	5.32 (0.86, 13.54)	1.29 (0.43,5.46)	−2.2 (-5.86, −0.63)	<0.001
MPRED (mg/d)	20 (20, 30)	15 (8, 22)	−5 (−18, 0)	<0.001

Data are presented as median (interquartile range). mMRC, modified Medical Research Council dyspnea scale; ESR, erythrocyte sedimentation rate; CRP, C-reactive protein; GGO, ground-glass opacity; HCs, Honeycomb cysts; HRCT, high-resolution computed tomography; MPRED, methylprednisolone; Boot CI, Bootstrap confidence interval (10,000 replicates). Median change is calculated as post-treatment value minus baseline value. p values were derived from Wilcoxon signed-rank tests.

### 3.3 Correlations among parameter changes after tacrolimus therapy

**FIGURE 2:**
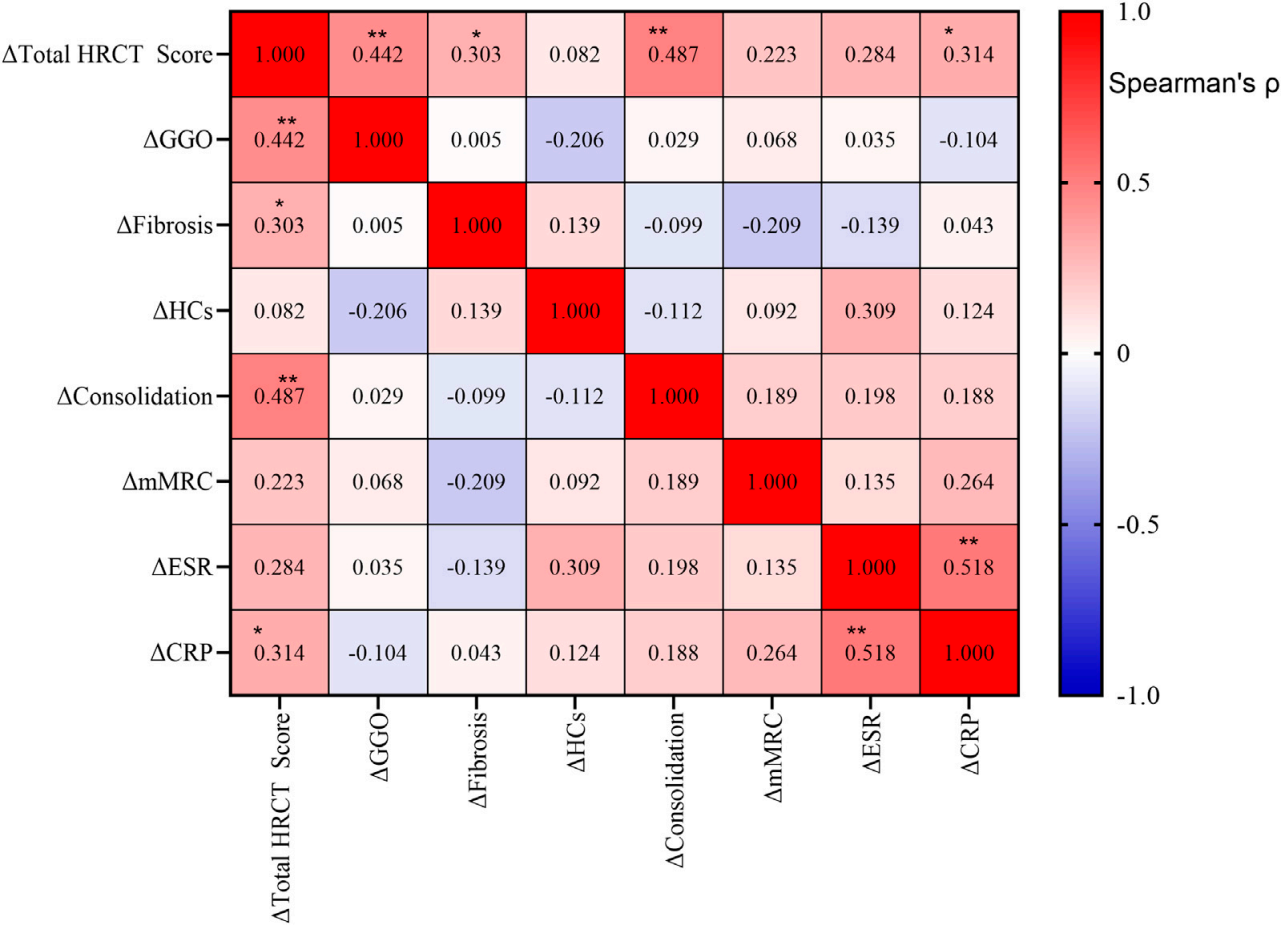
Correlations among changes in clinical and radiological parameters after treatment. Spearman correlation analysis was performed on the changes (Δ) from baseline in the total high-resolution computed tomography (HRCT) score, ground-glass opacity (GGO), fibrosis, honeycombing cysts (HCs), consolidation, modified Medical Research Council (mMRC) dyspnea scale, erythrocyte sedimentation rate (ESR), and C-reactive protein (CRP). The heatmap color intensity represents the strength and direction of the correlation coefficient (ρ), with red indicating positive correlations and blue indicating negative correlations. The exact ρ value is displayed in each cell. Statistical significance is indicated as follows: *P < 0.05; **P < 0.01.

Spearman correlation analysis revealed strong associations between radiographic and inflammatory markers following tacrolimus treatment (Figure 2). Total HRCT score improvement correlated most significantly with reduction in consolidation (ρ = 0.487, P < 0.001) and ground-glass opacities (ρ = 0.442, P = 0.002), and was also associated with decreases in CRP (ρ = 0.314, P = 0.030) and ESR (ρ = 0.284, P = 0.050). Consolidation reduction showed significant synchronization with CRP decline (ρ = 0.354, P = 0.023). In contrast, changes in fibrosis and HCs exhibited no significant correlations with other parameters. mMRC dyspnea grade improvement showed no statistical association with imaging or inflammatory markers. A strong concordance was observed between ESR and CRP changes (ρ = 0.518, P < 0.001).

### 3.4 Factors influencing treatment response to tacrolimus

To identify independent predictors of changes in the total HRCT score (Δ total HRCT score) following tacrolimus therapy in patients with IPAF, we performed a multivariable linear regression analysis. After adjustment, only the baseline consolidation score was significantly associated with improvement in the total HRCT score (B = −0.249, 95% CI: −0.420 to −0.078, p = 0.005; β = −0.397), indicating that patients with more severe consolidation at baseline exhibited greater radiographic improvement after treatment. Although not statistically significant, baseline GGO and smoking showed a tentative association with Δ total HRCT score. The baseline total CT score itself did not predict the degree of improvement. The model explained 22.8% of the variance in treatment response (adjusted *R* ^2^ = 0.228) (Table 3).

**TABLE 3:** Multiple linear regression analysis of factors associated with change in total HRCT score (ΔTotal HRCT score).

Variable	Unstandardized coefficient (B)	95% CI for B	Standardized coefficient (β)	t value	p value
Constant	−0.017	(-2.654, 2.620)	NA	−0.013	0.989
Baseline consolidation	−0.334	(-0.578, −0.090)	−0.424	−2.764​​	0.008
Baseline GGO	−0.274	(-0.575, 0.027)	−0.246	−1.830	0.074
Smoking	1.891	(-0.391, 4.173)	0.230	1.674	0.101
Baseline total HRCT score	0.002	(-0.171, 0.175)	0.003	0.021	0.983

The dependent variable is the change in total HRCT, score (Δ total HRCT, score). GGO, ground-glass opacity; HCs, Honeycomb cysts.

### 3.5 Association between autoantibody type and response to tacrolimus therapy

To investigate whether autoantibody type influences treatment response to tacrolimus in patients with IPAF, patients were categorized into two groups based on serological profile: the MSAs group (n = 28) and the MAAs group (n = 15). The two groups were comparable at baseline in terms of gender, age, smoking, radiographic findings, symptom scores, and levels of serum inflammatory markers (all p > 0.05) (Table 4).

**TABLE 4:** Comparison of baseline characteristics between patients with myositis-specific antibodies (MSAs) and myositis-associated antibodies (MAAs).

Variable	MSAs (n = 28)	MAAs (n = 15)	U	P value
Age, years	57 (50.5, 58)	55 (46, 59)	173.5	0.35
Male,n (%)	8 (28.6%)	4 (26.7%)	NA	1.00
Smoking history,n (%)	1 (3.6%)	2 (13.3%)	NA	0.535
Total HRCT score	16 (12.25, 21)	18 (12, 19)	209	0.98
GGO	4.5 (2, 6)	4 (2, 6)	191	0.625
Fibrosis	7 (5, 10)	10 (6, 12)	162.5	0.224
HCs	0 (0, 0)	0 (0, 0)	201	0.649
Consolidation	3 (0, 8)	2 (0, 5)	162.5	0.217
mMRC	3 (2, 3)	3 (2, 3)	191.5	0.601
0, n (%)	0	0	NA	NA
1, n (%)	3 (10.7%)	1 (6.7%)	NA	NA
2, n (%)	10 (35.7%)	4 (26.7%)	NA	NA
3, n (%)	13 (46.4%)	10 (66.7%)	NA	NA
4, n (%)	2 (7.1%)	0	NA	NA
ESR, mm/h	26 (12, 46)	16 (11, 29)	168.5	0.29
CRP, mg/L	4.97 (1.05, 11.77)	1 (0.37, 16.66)	193.5	0.674
MPRED, mg/d	25 (20, 34)	20 (10, 30)	102.5	0.062

Variables were presented as median (interquartile range) or N (%). mMRC, modified Medical Research Council dyspnea scale; ESR, erythrocyte sedimentation rate; CRP, C-reactive protein; GGO, ground-glass opacity; HRCT, high-resolution computed tomography; MPRED, methylprednisolone; HCs, Honeycomb cysts; NA, not applicable/not available.

Intra-group analyses (Table 5) revealed that patients in the MSAs group exhibited significant improvements following tacrolimus treatment across multiple domains: total HRCT score (p < 0.001), GGO score (p = 0.008), consolidation score (p < 0.001), mMRC grade (p < 0.001), ESR (p < 0.001), CRP (p < 0.001), and MPRED dosage (p < 0.001) were all significantly reduced. In contrast, the MAAs group showed significant improvements only in consolidation (p = 0.024), mMRC grade (p = 0.002), ESR (p = 0.004), and MPRED dosage (p = 0.012). Key radiographic parameters in this group—including total HRCT score, GGO, and fibrosis scores—did not demonstrate statistically significant improvement (p = 0.115, 0.162, and 0.172, respectively). Furthermore, the change in CRP level in the MAAs group did not reach statistical significance (p = 0.096) (Table 5).

**TABLE 5:** Comparison of treatment response to tacrolimus between and within MSAs and MAAs groups.

Variable	MSAs (n = 28)			MAAs (n = 15)			P3
	Baseline	Post-treatment	P1	Baseline	Post-treatment	P2	
Total HRCT score	16 (12.25, 21)	14 (9.25, 19)	<0.001	18 (12, 19)	15 (9, 20)	0.115	0.302
GGO	4.5 (2, 6)	3 (1, 5.5)	0.008	4 (2, 6)	−2 (2, 6)	0.162	0.948
Fibrosis	7 (5, 10)	7.5 (5, 10)	0.619	10 (6, 12)	8 (5, 12)	0.172	0.497
HCs	0 (0, 0)	0 (0, 0)	0.063	0 (0, 0)	0 (0, 0)	0.317	0.522
Consolidation	3 (0, 8)	1 (0, 4)	<0.001	2 (0, 5)	0 (0, 2)	0.024	0.386
mMRC grade	3 (2, 3)	1 (1, 1)	<0.001	3 (2, 3)	1 (1, 1)	0.002	0.881
ESR (mm/h)	4.97 (1.05, 11.77)	1.33 (0.38, 4.86)	<0.001	16 (11, 29)	10 (3, 12)	0.004	0.949
CRP (mg/L)	16 (12.25, 21)	14 (9.25, 19)	<0.001	1 (0.37, 16.66)	0.83 (0.39, 6.20)	0.096	0.273
MPRED (mg/d)	25 (20, 34)	14 (7.5, 20)	<0.001	20 (10, 30)	10 (7.88, 28.13)	0.012	0.022

MSAs, myositis-specific antibodies group (n = 28); MAAs, myositis-associated antibodies group (n = 15); HRCT, computed tomography; GGO, Ground-glass opacity; HCs, Honeycomb cysts; mMRC, modified Medical Research Council dyspnea scale; ESR, erythrocyte sedimentation rate; CRP, C-reactive protein; MPRED, Methylprednisolone. Data are presented as median (first quartile, third quartile). Within-group comparisons (MSAs, P_1_; MAsA, P_2_) were analyzed using Wilcoxon signed-rank tests. Between-group comparisons of Δ values (post-treatment minus baseline, P_3_) were performed using Mann-Whitney U tests.

### 3.6 Adverse events

Among the 48 patients treated with tacrolimus and glucocorticoids in this study, all observed adverse events were non-fatal and did not lead to treatment discontinuation or dose adjustment. Hypomagnesemia was the most frequently reported adverse event (n = 27, 56.25%), and all affected patients achieved normalized magnesium levels following oral supplementation. Infections occurred in 19 patients (39.58%); all cases were effectively managed during hospitalization, and no life-threatening events were reported. However, as MPRED are also associated with infectious complications, the contribution of tacrolimus alone could not be definitively established. No cases of renal insufficiency were observed during the study period. Gastrointestinal discomfort and hypertension were documented, though attribution specifically to tacrolimus was confounded by underlying disease activity and other potential factors.

## 4 Discussion

In this study, we comprehensively evaluated the therapeutic efficacy of tacrolimus in patients with IPAF from three dimensions—clinical symptoms, serological markers, and imaging manifestations—using a combination of ESR, CRP, mMRC grade, and chest HRCT scores. The results demonstrated significant improvements in most patients across these parameters compared to pre-treatment levels. Due to the presence of severe pulmonary involvement and dyspnea in a considerable proportion of enrolled patients, standardized pulmonary function tests could not be reliably performed, resulting in missing baseline pulmonary function data in 32.3% of cases. Therefore, to avoid significant bias that may arise from incomplete data, we have prudently decided not to include pulmonary function results in this statistical analysis.

The selection of tacrolimus as the immunosuppressive regimen in this study must be interpreted within its specific clinical context. We fully acknowledge that, according to international guideline consensus, tacrolimus is not the standard first-line treatment for most interstitial lung diseases (Maher, 2024). However, the patients enrolled in this study predominantly exhibited rapid disease progression or poor response to initial immunosuppressive therapies (such as glucocorticoids, mycophenolate mofetil, etc.), presenting limitations in clinical treatment options. Within this therapeutic dilemma, the selection of tacrolimus represents an individualized treatment decision primarily based on its potent immunosuppressive properties and the therapeutic potential observed with this agent in other autoimmune-associated diffuse lung diseases (e.g., dermatomyositis/polymyositis-associated interstitial lung disease, anti-synthetase syndrome-associated interstitial lung disease) (Wilkes et al., 2005; Takada, Nagasaka, and Miyasaka, 2005; Takada et al., 2020). This study aims to explore alternative therapeutic strategies that may effectively control disease progression when conventional treatments prove insufficient. Therefore, these findings should not be interpreted as supporting the use of tacrolimus for all IPAF patients. Instead, they provide preliminary exploratory data on the efficacy and safety of this drug for refractory or severe IPAF patients, laying the groundwork for future in-depth studies in specific subgroups.

Our findings confirm that tacrolimus treatment significantly ameliorates active lung lesions (consolidation and GGO) and systemic inflammation in IPAF patients. Improvement in the total HRCT score was primarily attributable to the resolution of GGO and consolidation. These radiographic features are often associated with “alveolitis” in IPAF, representing reversible disease processes (Goldin et al., 2008). Consistent with our results, a study by McCoy et al. (2018) on MMF in IPAF also reported that patients with predominant GGO and minimal fibrosis responded better to treatment (McCoy et al., 2018). Histologically, consolidation and GGO often correspond to NSIP and OP, characterized by intra-alveolar fibroblastic plugs and lymphocytic infiltration—particularly of T cells (Bankier et al., 2024; American Thoracic Society/European Respiratory Society International Multidisciplinary Consensus Classification of the Idiopathic Interstitial Pneumonias, 2002; Joerns et al., 2022; Qin et al., 2013). Tacrolimus is believed to modulate immune responses primarily through its interaction with intracellular FKBP-12, forming a complex that inhibits calcineurin phosphatase activity. This inhibition is thought to reduce the production of key cytokines, including IL-2, ultimately leading to suppression of T-cell activation and proliferation (Takada et al., 2005). The significant improvement in mMRC grade and successful reduction in MPRED dosage observed in this study further support the clinical benefits of tacrolimus.

Multivariate analysis identified the baseline consolidation score as an independent predictor of improvement in the total HRCT score (B = −0.249, p = 0.005). Patients with more severe baseline consolidation exhibited greater radiographic improvement, possibly reflecting a more concentrated acute inflammatory response that is particularly amenable to tacrolimus-mediated immunomodulation (Bankier et al., 2024; American Thoracic Society/European Respiratory Society International Multidisciplinary Consensus Classification of the Idiopathic Interstitial Pneumonias, 2002). This finding suggests that baseline consolidation may serve as an imaging biomarker for identifying patients most likely to benefit from treatment in future prospective studies. Additionally, although some patients showed progression of HCs after tacrolimus therapy, with no significant change in fibrosis, these observations indicate that while tacrolimus is effective against active exudative lesions, it has limited efficacy against established structural damage such as fibrosis and honeycombing.

Recent multicenter research by Graham et al. highlighted that IPAF patients with different autoantibody types exhibit distinct clinical features, treatment responses, and prognoses. Those with MSAs tend to be younger, exhibit non-UIP patterns on HRCT, and demonstrate greater sensitivity to immunosuppressive therapy (e.g., MMF and corticosteroids) (Graham et al., 2020). In our cohort, although median scores for GGO and consolidation were higher in the MSAs subgroup, while fibrosis scores were lower compared to the MAAs subgroup, these differences did not reach statistical significance. Following tacrolimus treatment, the MSAs subgroup showed significant improvements across all evaluated parameters, whereas the MAAs subgroup improved only in certain measures (consolidation, mMRC grade, ESR, and MPRED dosage), with no significant changes in key imaging parameters (total HRCT score, GGO, fibrosis) or CRP. These differences may reflect distinct immunopathological mechanisms: MSAs (e.g., antisynthetase antibodies) are often linked to inflammatory phenotypes and favorable responses to immunosuppression, while MAAs may involve more complex or overlapping disease processes (Graham et al., 2020; Yamano et al., 2023). Although limited by sample size and requiring cautious interpretation, these subgroup results strongly suggest substantial heterogeneity within IPAF, underscoring the need for future treatment strategies tailored according to antibody profile.

Tacrolimus demonstrated an acceptable safety profile in this IPAF cohort. Most adverse events, including hypomagnesemia, were manageable with supportive care. Clinical monitoring of serum magnesium and signs of infection, particularly during concomitant glucocorticoid therapy, is recommended. Larger studies with longer follow-up are needed to further establish its long-term safety.

As a single-center retrospective analysis, this study has several limitations. The sample size was limited, particularly in the MAAs subgroup (n = 15), which may have reduced statistical power. Non-standardized treatment protocols and follow-up durations may have introduced selection bias. Furthermore, the retrospective design limited the ability to control fully for confounding variables. Future multi-center, prospective studies with larger sample sizes are warranted to validate the efficacy of tacrolimus and explore its potential value in combination with antifibrotic agents.

## 5 Conclusion

This study demonstrates that tacrolimus significantly improves clinical symptoms, active pulmonary lesions, and systemic inflammation in IPAF, and facilitates glucocorticoid tapering. However, it has limited effect on reversing fibrotic changes. Baseline consolidation may serve as a predictive imaging biomarker for treatment response, and patients with MSAs appear to exhibit a more comprehensive response compared to those with MAAs. These findings provide a foundation for developing precision treatment strategies based on antibody subtype and imaging characteristics.

## Data Availability

The original contributions presented in the study are included in the article/supplementary material, further inquiries can be directed to the corresponding author.

## Abbreviations

ILD, interstitial lung disease; IIP, idiopathic interstitial pneumonia; CTD, connective tissue disease; IPF, idiopathic pulmonary fibrosis; ANA, antinuclear antibody; CCP, cyclic citrullinated peptide; HRCT, high-resolution computed tomography; IPAF, interstitial pneumonia with autoimmune features; LIP, lymphocytic interstitial pneumonia; NSIP, nonspecific interstitial pneumonia; OP, organizing pneumonia; RF, rheumatoid factor; mMRC, modified Medical Research Council dyspnea scale; ESR, erythrocyte sedimentation rate; CRP, C-reactive protein; GGO, ground-glass opacity; HCs, Honeycomb cysts; MPRED, Methylprednisolone; SSc-ILD, systemic sclerosis-associated interstitial lung disease; MAAs, myositis-associated antibodies; MSAs, myositis-specific antibodies; IIM, inflammatory myopathy; MMF, mycophenolate mofetil; ERS/ATS, European Respiratory Society/American Thoracic Society.
